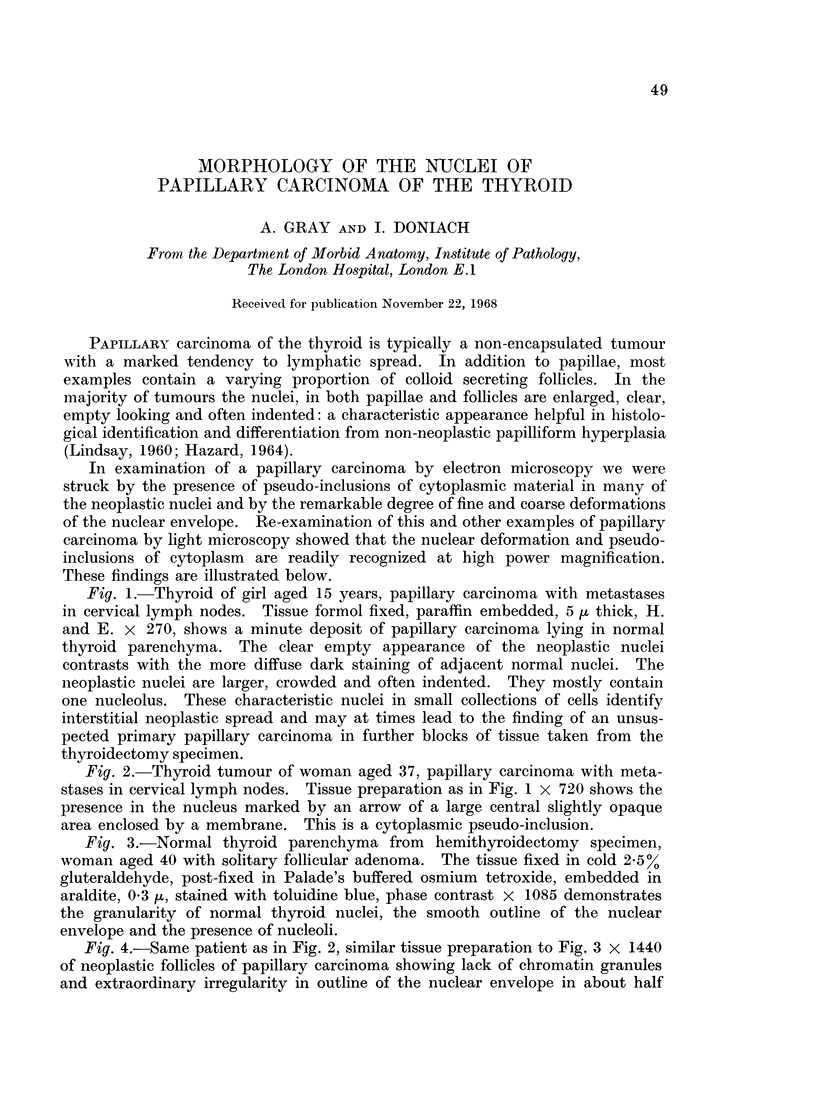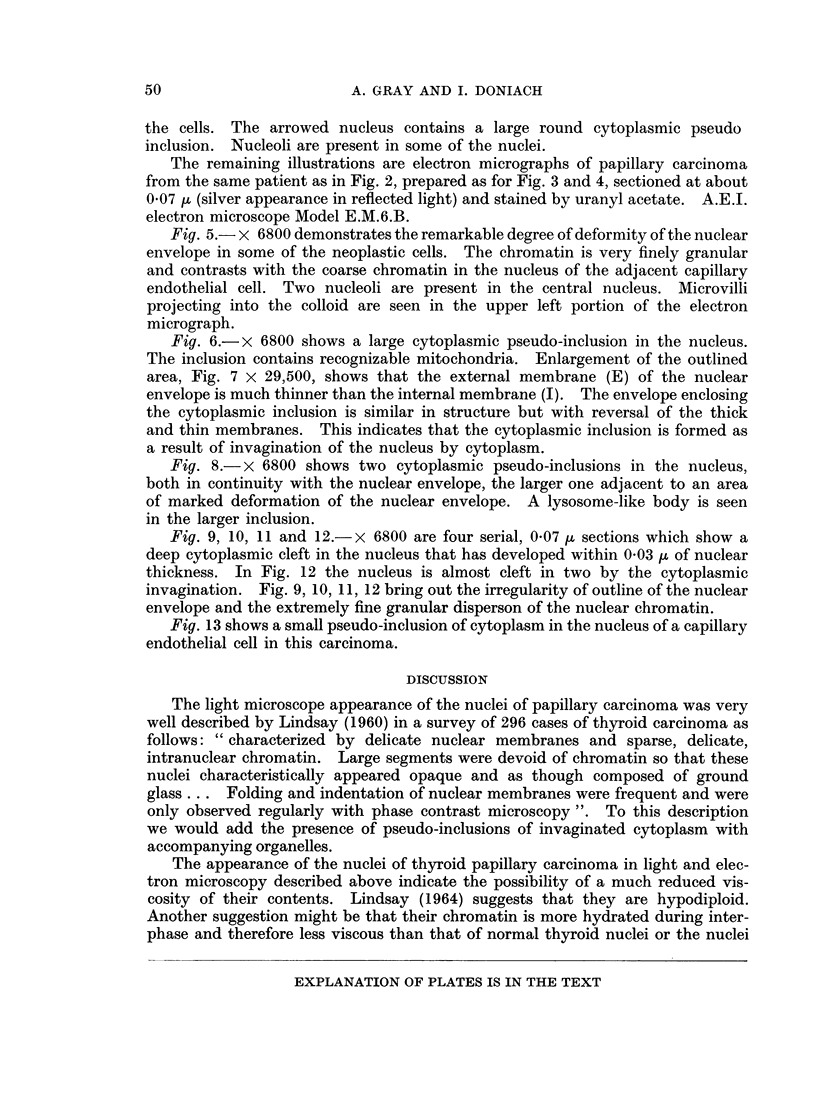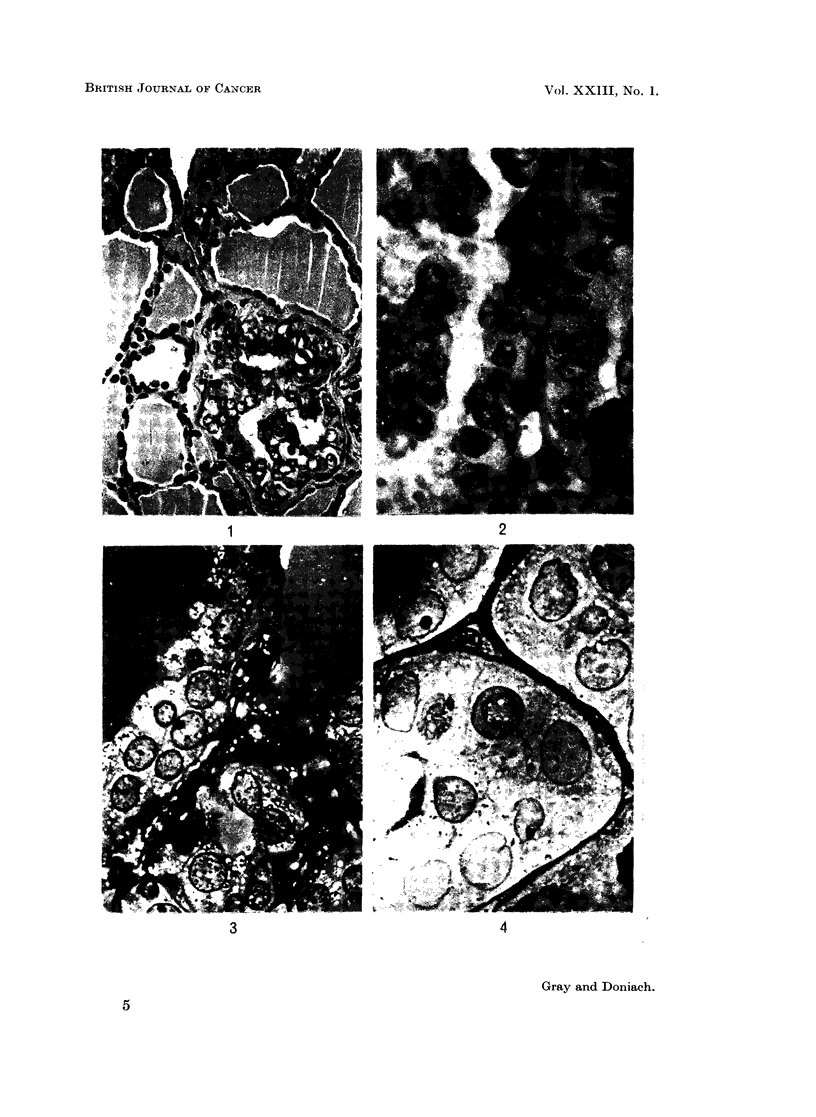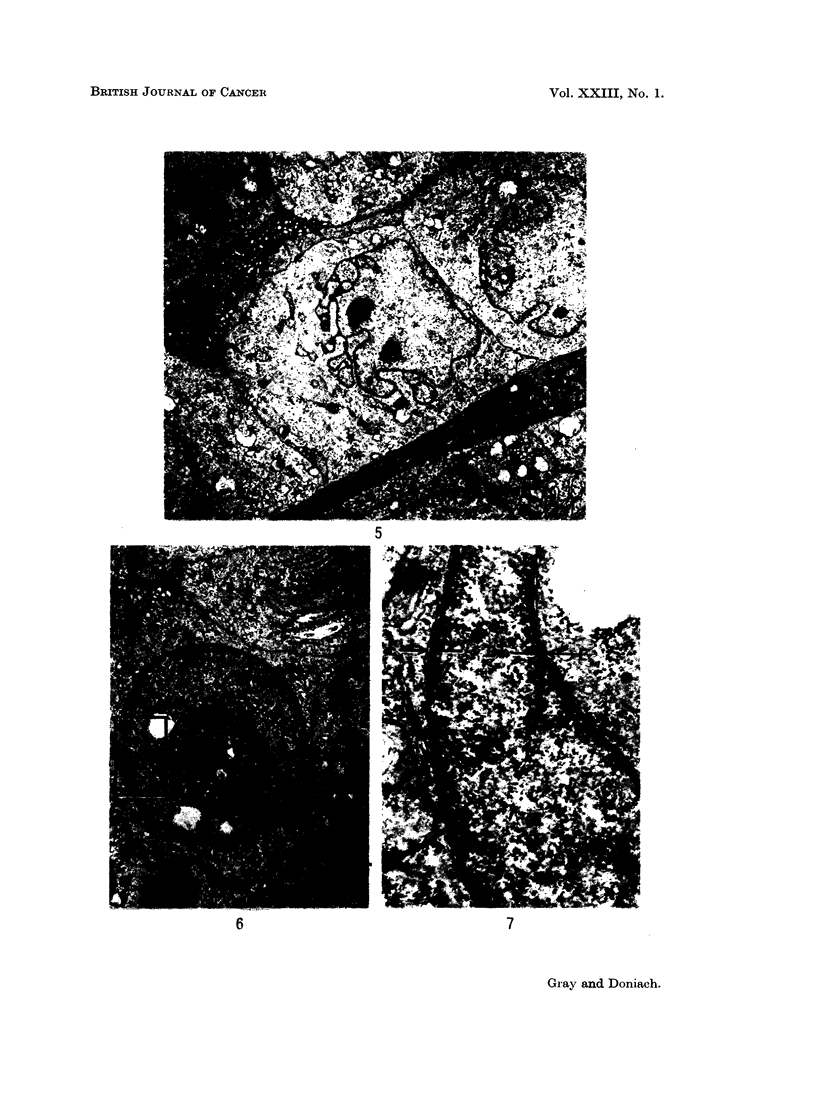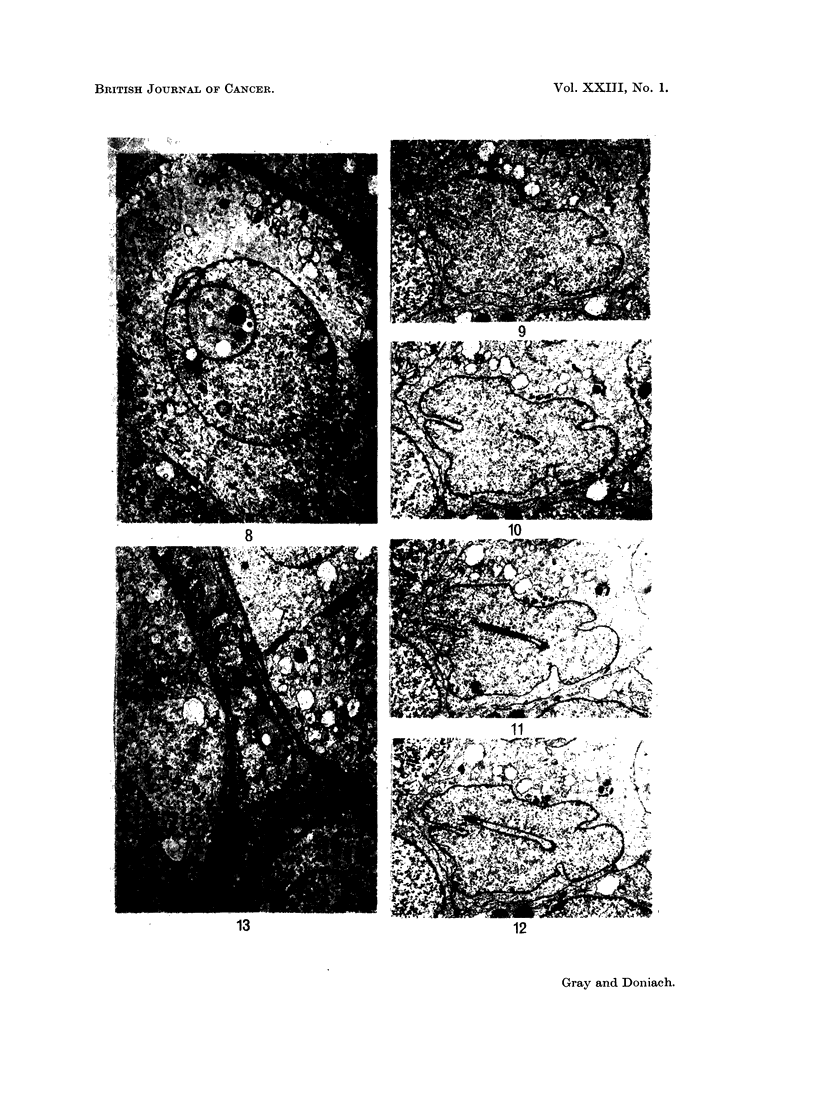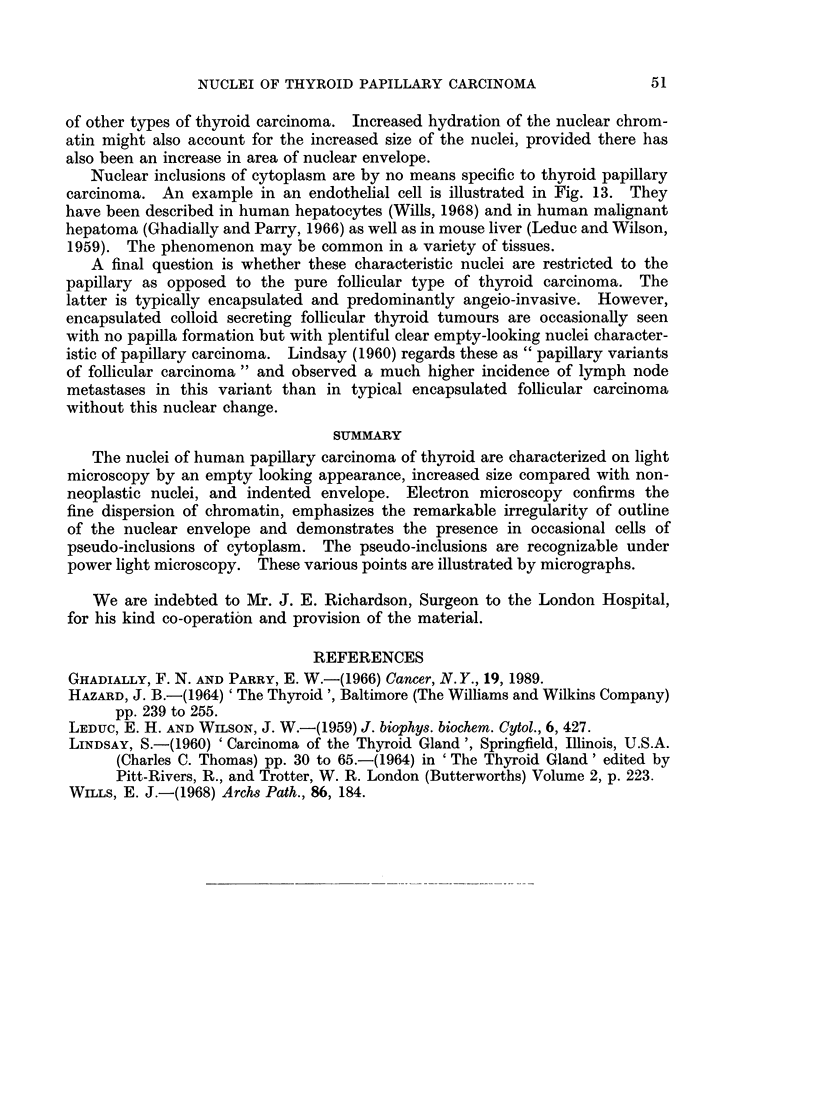# Morphology of the nuclei of papillary carcinoma of the thyroid.

**DOI:** 10.1038/bjc.1969.8

**Published:** 1969-03

**Authors:** A. Gray, I. Doniach

## Abstract

**Images:**


### MORPHOLOGY OF THE NUCLEI OF PAPILLARY CARCINOMA OF THE THYROID

A. GRAY AND I. DONIACH

*From the Department of Morbid Anatomy, Institute of Pathology, The London Hospital, London E.*1

Received for publication November 22, 1968

PAPILLARY carcinoma of the thyroid is typically a non-encapsulated tumour with a marked tendency to lymphatic spread. In addition to papillae, most examples contain a varying proportion of colloid secreting follicles. In the majority of tumours the nuclei, in both papillae and follicles are enlarged, clear, empty looking and often indented: a characteristic appearance helpful in histological identification and differentiation from non-neoplastic papilliform hyperplasia (Lindsay, 1960; Hazard, 1964).

In examination of a papillary carcinoma by electron microscopy we were struck by the presence of pseudo-inclusions of cytoplasmic material in many of the neoplastic nuclei and by the remarkable degree of fine and coarse deformations of the nuclear envelope. Re-examination of this and other examples of papillary carcinoma by light microscopy showed that the nuclear deformation and pseudo-inclusions of cytoplasm are readily recognized at high power magnification. These findings are illustrated below.

*Fig.* 1.—Thyroid of girl aged 15 years, papillary carcinoma with metastases in cervical lymph nodes. Tissue formol fixed, paraffin embedded, 5 $\mu$ thick, H. and E. $\times$ 270, shows a minute deposit of papillary carcinoma lying in normal thyroid parenchyma. The clear empty appearance of the neoplastic nuclei contrasts with the more diffuse dark staining of adjacent normal nuclei. The neoplastic nuclei are larger, crowded and often indented. They mostly contain one nucleolus. These characteristic nuclei in small collections of cells identify interstitial neoplastic spread and may at times lead to the finding of an unsuspected primary papillary carcinoma in further blocks of tissue taken from the thyroidectomy specimen.

*Fig.* 2.—Thyroid tumour of woman aged 37, papillary carcinoma with metastases in cervical lymph nodes. Tissue preparation as in Fig. 1 $\times$ 720 shows the presence in the nucleus marked by an arrow of a large central slightly opaque area enclosed by a membrane. This is a cytoplasmic pseudo-inclusion.

*Fig.* 3.—Normal thyroid parenchyma from hemithyroidectomy specimen, woman aged 40 with solitary follicular adenoma. The tissue fixed in cold 2·5% gluteraldehyde, post-fixed in Palade's buffered osmium tetroxide, embedded in araldite, 0·3 $\mu$, stained with toluidine blue, phase contrast $\times$ 1085 demonstrates the granularity of normal thyroid nuclei, the smooth outline of the nuclear envelope and the presence of nucleoli.

*Fig.* 4.—Same patient as in Fig. 2, similar tissue preparation to Fig. 3 $\times$ 1440 of neoplastic follicles of papillary carcinoma showing lack of chromatin granules and extraordinary irregularity in outline of the nuclear envelope in about half the cells. The arrowed nucleus contains a large round cytoplasmic pseudo inclusion. Nucleoli are present in some of the nuclei.

The remaining illustrations are electron micrographs of papillary carcinoma from the same patient as in Fig. 2, prepared as for Fig. 3 and 4, sectioned at about 0·07 $\mu$ (silver appearance in reflected light) and stained by uranyl acetate. A.E.I. electron microscope Model E.M.6.B.

*Fig.* 5.—$\times$ 6800 demonstrates the remarkable degree of deformity of the nuclear envelope in some of the neoplastic cells. The chromatin is very finely granular and contrasts with the coarse chromatin in the nucleus of the adjacent capillary endothelial cell. Two nucleoli are present in the central nucleus. Microvilli projecting into the colloid are seen in the upper left portion of the electron micrograph.

*Fig.* 6.—$\times$ 6800 shows a large cytoplasmic pseudo-inclusion in the nucleus. The inclusion contains recognizable mitochondria. Enlargement of the outlined area, Fig. 7 $\times$ 29,500, shows that the external membrane (E) of the nuclear envelope is much thinner than the internal membrane (I). The envelope enclosing the cytoplasmic inclusion is similar in structure but with reversal of the thick and thin membranes. This indicates that the cytoplasmic inclusion is formed as a result of invagination of the nucleus by cytoplasm.

*Fig.* 8.—$\times$ 6800 shows two cytoplasmic pseudo-inclusions in the nucleus, both in continuity with the nuclear envelope, the larger one adjacent to an area of marked deformation of the nuclear envelope. A lysosome-like body is seen in the larger inclusion.

*Fig.* 9, 10, 11 and 12.—$\times$ 6800 are four serial, 0·07 $\mu$ sections which show a deep cytoplasmic cleft in the nucleus that has developed within 0·03 $\mu$ of nuclear thickness. In Fig. 12 the nucleus is almost cleft in two by the cytoplasmic invagination. Fig. 9, 10, 11, 12 bring out the irregularity of outline of the nuclear envelope and the extremely fine granular disperson of the nuclear chromatin.

*Fig.* 13 shows a small pseudo-inclusion of cytoplasm in the nucleus of a capillary endothelial cell in this carcinoma.

### DISCUSSION

The light microscope appearance of the nuclei of papillary carcinoma was very well described by Lindsay (1960) in a survey of 296 cases of thyroid carcinoma as follows: " characterized by delicate nuclear membranes and sparse, delicate, intranuclear chromatin. Large segments were devoid of chromatin so that these nuclei characteristically appeared opaque and as though composed of ground glass . . . Folding and indentation of nuclear membranes were frequent and were only observed regularly with phase contrast microscopy ". To this description we would add the presence of pseudo-inclusions of invaginated cytoplasm with accompanying organelles.

The appearance of the nuclei of thyroid papillary carcinoma in light and electron microscopy described above indicate the possibility of a much reduced viscosity of their contents. Lindsay (1964) suggests that they are hypodiploid. Another suggestion might be that their chromatin is more hydrated during interphase and therefore less viscous than that of normal thyroid nuclei or the nuclei

EXPLANATION OF PLATES IS IN THE TEXT

1 2 3 4

Gray and Doniach.

5

6

7

Gray and Doniach.

of other types of thyroid carcinoma. Increased hydration of the nuclear chromatin might also account for the increased size of the nuclei, provided there has also been an increase in area of nuclear envelope.

Nuclear inclusions of cytoplasm are by no means specific to thyroid papillary carcinoma. An example in an endothelial cell is illustrated in Fig. 13. They have been described in human hepatocytes (Wills, 1968) and in human malignant hepatoma (Ghadially and Parry, 1966) as well as in mouse liver (Leduc and Wilson, 1959). The phenomenon may be common in a variety of tissues.

A final question is whether these characteristic nuclei are restricted to the papillary as opposed to the pure follicular type of thyroid carcinoma. The latter is typically encapsulated and predominantly angeio-invasive. However, encapsulated colloid secreting follicular thyroid tumours are occasionally seen with no papilla formation but with plentiful clear empty-looking nuclei characteristic of papillary carcinoma. Lindsay (1960) regards these as " papillary variants of follicular carcinoma " and observed a much higher incidence of lymph node metastases in this variant than in typical encapsulated follicular carcinoma without this nuclear change.

### SUMMARY

The nuclei of human papillary carcinoma of thyroid are characterized on light microscopy by an empty looking appearance, increased size compared with non-neoplastic nuclei, and indented envelope. Electron microscopy confirms the fine dispersion of chromatin, emphasizes the remarkable irregularity of outline of the nuclear envelope and demonstrates the presence in occasional cells of pseudo-inclusions of cytoplasm. The pseudo-inclusions are recognizable under power light microscopy. These various points are illustrated by micrographs.

We are indebted to Mr. J. E. Richardson, Surgeon to the London Hospital, for his kind co-operation and provision of the material.

### REFERENCES

Ghadially, F. N. and Parry, E. W.—(1966) *Cancer, N.Y.*, **19**, 1989.

Hazard, J. B.—(1964) ' The Thyroid ', Baltimore (The Williams and Wilkins Company) pp. 239 to 255.

Leduc, E. H. and Wilson, J. W.—(1959) *J. biophys. biochem. Cytol.*, **6**, 427.

Lindsay, S.—(1960) ' Carcinoma of the Thyroid Gland ', Springfield, Illinois, U.S.A. (Charles C. Thomas) pp. 30 to 65.—(1964) in ' The Thyroid Gland ' edited by Pitt-Rivers, R., and Trotter, W. R. London (Butterworths) Volume 2, p. 223.

Wills, E. J.—(1968) *Archs Path.*, **86**, 184.